# A Review on Asthma and Allergy: Current Understanding on Molecular Perspectives

**DOI:** 10.3390/jcm13195775

**Published:** 2024-09-27

**Authors:** Gassem Gohal, Sivakumar S. Moni, Mohammed Ali Bakkari, Mohamed Eltaib Elmobark

**Affiliations:** 1 Department of Pediatrics, Faculty of Medicine, Jazan University, Jazan 45142, Saudi Arabia; ggohal@jazanu.edu.sa; 2 Health Research Centre, Jazan University, Jazan 45142, Saudi Arabia; mohamedeltaib.me@gmail.com; 3 Department of Pharmaceutics, College of Pharmacy, Jazan University, Jazan 45142, Saudi Arabia; mbakkari@jazanu.edu.sa; 4 Department of Clinical Pharmacy, College of Pharmacy, Jazan University, Jazan 45142, Saudi Arabia

**Keywords:** asthma, allergic biomarkers, autoimmunity, dysregulated immune system, cytokine network, mechanistic approach

## Abstract

Asthma, a complex disease characterized by persistent airway inflammation, remains an urgent global health concern. We explored the critical role of allergic biomarkers and dysregulated immune system in asthma through an extensive literature review in databases such as Web of Science, PubMed, EMBASE, Scopus, and Google Scholar. This review summarizes the growing data on the pivotal role of allergic biomarkers and dysregulated immune system in the development and evolution of asthma. Recent studies have uncovered several biomarkers that elucidate intrinsic allergic mechanisms in individuals with asthma. This article highlights these biomarkers’ potential in predicting asthma onset, assessing its intensity, guiding therapeutic interventions, and tracking disease progression. We also explore the innovative therapeutic prospects arising from the convergence of allergy and dysregulated immune system in asthma and emphasize the potential for precision medicine approaches. Understanding allergic biomarkers intertwined with a dysregulated immune system heralds a new era in asthma treatment and points to improved and individualized treatment modalities.

## 1. Introduction

**Figure 1:**
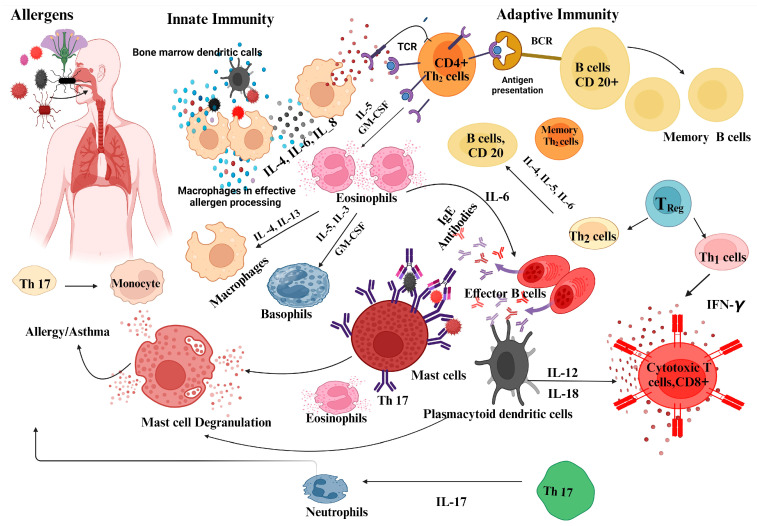
Immune mechanism in allergic asthma.

Asthma is a persistent inflammation of the airways that leads to symptoms such as wheezing, shortness of breath, coughing, and tightness in the chest. The exact causes of asthma remain elusive, but it is known that both genetic predisposition and environmental triggers contribute to the onset of the disease [1]. Allergic and autoimmune inflammatory reactions are two mechanisms that may contribute to the development and progression of asthma (Figure 1). Increased IgE levels are frequently observed in people with allergic asthma, as noted by Wang and colleagues in 2023 [2]. IgE is instrumental in allergic responses, as it binds to allergenic substances, releasing substances that cause inflammation in the airways and tightening the bronchial passages. Eosinophils are involved in the immune response against parasites and allergic reactions. In asthma, an increased concentration of eosinophils is often observed in the blood, sputum, or bronchial biopsies. Eosinophils release inflammatory substances that contribute to airway inflammation and remodeling. Nitric oxide is produced by cells in the airways, including eosinophils, during inflammation [3]. Measuring fractional exhaled nitric oxide (FeNO) levels can help assess airway inflammation, particularly eosinophil inflammation, and monitor response to treatment in asthma patients [4]. The use of FeNO as a biomarker for airway inflammation in asthma is associated with certain limitations, particularly with regard to false positive results. Elevated FeNO levels can also occur in conditions other than asthma and can lead to false-positive results. For example, individuals with allergic rhinitis, eosinophilic bronchitis, or even environmental factors such as smoking, or air pollution may have high FeNO levels even though they do not have asthma [4,5,6].

Dysregulation of the immune system in asthma is a disturbance of normal immune homeostasis that leads to an imbalance of immune responses. This dysregulation often manifests itself in an exaggerated immune response characterized by hyperactivation of immune cells, excessive production of inflammatory cytokines, overlap between allergic markers, and increased airway hyperreactivity. However, it can also lead to an inadequate or inappropriate immune response, which impairs the body’s ability to regulate inflammation and leads to chronic disease progression. This complex interplay of immune pathways is the basis of asthma pathogenesis [7]. Although asthma is primarily a chronic inflammatory disorder, there is evidence that dysregulated immune system mechanisms may also contribute to the development and progression of the disease [8,9]. In asthma, a dysregulated immune system can cause the immune system to target and attack components of the respiratory system mistakenly. Some people with asthma can produce autoantibodies that recognize and bind to self-antigens in the respiratory system [7]. By driving immune system dysregulation and promoting persistent inflammation, they contribute to the chronicity and severity of the disease, which is more difficult to treat with conventional asthma treatments [10].

T helper cells play a crucial role in regulating immune responses. There is evidence of an imbalance of T helper cells in asthma, particularly a shift toward dominance of Th_2_ cells [11]. Th_2_ cells produce cytokines that promote allergic inflammation, leading to airway hyperreactivity and asthma symptoms. Regulatory T cells (Tregs) are a specific group of T cells that help maintain the immune system’s balance and prevent excessive immune responses. Dysfunction or decreased numbers of Tregs have been observed in asthma patients, indicating impaired immune regulation [12]. This may contribute to excessive immune responses against harmless substances, such as allergens, leading to asthma symptoms. The cytokine network is of utmost importance in controlling the immune response in autoimmune asthma. Cytokines, tiny proteins released by diverse immune cells, serve as messengers that enable cell communication and orchestrate immune reactions. 

IL-4 is produced by Th_2_ cells and plays a central role in promoting the production of IgE antibodies involved in allergic reactions. In asthma, elevated levels of IL-4 contribute to the activation of Th_2_ responses, leading to increased production of IgE and recruitment of eosinophils [13,14]. IL-5 plays a central role in the differentiation, activation, and survival of eosinophils, a specific type of white blood cell involved in allergic inflammation. In autoimmune asthma, IL-5 promotes the immigration of eosinophils into the airways, leading to tissue damage and worsening asthma symptoms [15]. 

IL-13 is another important cytokine produced by Th_2_ cells. It shares several biological activities with IL-4 and contributes to the pathology of autoimmune inflammatory reactions in asthma. IL-13 promotes airway hyperresponsiveness, mucus production, and eosinophil recruitment [16]. A subset of T cells mainly produces IL-17, called Th17 cells. In autoimmune inflammatory reactions in asthma, IL-17 promotes the recruitment and activation of neutrophils, another type of white blood cell. Neutrophils contribute to airway inflammation and tissue damage. TNF-α is a proinflammatory cytokine produced by various immune cells. It promotes airway inflammation, mucus production, and airway remodeling in autoimmune inflammatory reactions in asthma [17]. 

These and other cytokines interact and modulate the immune response in autoimmune inflammatory reactions in asthma, resulting in chronic airway inflammation, bronchial constriction, and other characteristic features of the disease. The cytokine network in autoimmune inflammatory reactions in asthma is complex and involves dysregulation of Th_2_ and Th_17_ responses, leading to persistent inflammation and tissue damage in the airways. The presence of allergic biomarkers and dysregulated immune system reactions in asthma may vary from person to person, and further research is needed to understand their role in the disease entirely. Table 1 overviews critical allergic markers commonly associated with asthma, outlining their specific roles in the asthmatic condition. This comprehensive review highlights the fascinating intersection between allergic markers in asthma and autoimmunity. By understanding the common immunologic mechanisms and potential clinical implications, researchers and clinicians can gain valuable insights into these complex diseases’ pathogenesis, diagnosis, and treatment. 

## 2. Inflammation and Asthma 

Inflammation is a critical factor in asthma, a long-term respiratory condition marked by swelling and constriction of the air passages. Exposure to specific stimuli like allergens, irritants, or respiratory infections can prompt an immune reaction in asthma patients, leading to inflamed airways. The inflammatory response in asthma involves several components. First, immune cells, particularly mast cells and eosinophils, release chemical messengers, including histamine, leukotrienes, and cytokines. These substances cause blood vessels to dilate, mucus production to increase, and smooth muscle surrounding the airways to contract, resulting in airway constriction, and breathing difficulties. Inflammation in asthma is classified as a type of chronic inflammation known as eosinophilic and neutrophilic inflammation. Eosinophils have a crucial role in allergic reactions. They infiltrate the tissues of the respiratory tract and release additional inflammatory substances that continue the inflammatory cascade and contribute to further damage to the respiratory tract [27]. The persistent inflammation in asthma can lead to airway wall remodeling over time. This remodeling includes structural changes such as thickening of the airway walls, enlargement of the mucous glands, and increased collagen deposition. These changes further contribute to airway narrowing and decreased lung function [28]. Treatment for asthma focuses on reducing inflammation and preventing or relieving symptoms. Commonly used medications include inhaled corticosteroid drugs that help reduce airway inflammation. T helper cells have an essential function in modulating immunological responses. Other medications, bronchodilators, leukotriene modifiers, and monoclonal antibodies that target specific inflammatory components may also be recommended, depending on the severity and treatment of the asthma. 

## 3. Allergies and Asthma

Allergies arise when the immune system responds to materials known as allergens, even though they are typically benign for many individuals. Pollen, dust mites, animal dander, animal saliva, specific foods, and insect bites are among the prevalent allergens. When an individual with allergies encounters an allergen, their immune system discharges substances such as histamine, resulting in allergic reactions. Symptoms of allergies can vary depending on the type of allergen and the individual, but they commonly include sneezing, itching, runny or stuffy nose, watery eyes, and skin rashes. Allergic reactions can range from mild to severe and, in some cases, can even be life-threatening anaphylaxis. Asthma and allergies are closely related, and individuals with asthma often have allergic triggers that can worsen their symptoms. Many people with asthma also have allergies, and the presence of allergies can worsen asthma symptoms. When an individual with allergic asthma meets an allergen, it can trigger an asthma attack or exacerbate existing asthma symptoms. The allergic response in the airways can cause increased inflammation, swelling, and mucus production, further narrowing the air passages and making it harder to breathe [29]. Managing allergies is an essential part of controlling allergic asthma. In Saudi Arabia, as in other regions of the world, asthma patients frequently suffer from allergies. The rate of allergic asthma is particularly high in this country. Several research projects have attempted to determine the extent of asthma and its allergic variant in different parts of Saudi Arabia [30,31]. Al Ghobain et al. (2018) conducted a cross-sectional survey using the European Community Respiratory Health Survey (ECRHS) questionnaire, in which 2405 participants took part. The prevalence of wheezing in the last 12 months without a cold was 18.2%, with no significant gender differences (*p* = 0.107). Physician-diagnosed asthma was reported by 11.3% of participants, again with no significant difference between men and women (*p* = 0.239). In addition, 10.6% of participants stated that they were taking medication for asthma. There were no significant differences between asthmatics and non-asthmatics in terms of area of residence (*p* = 0.07), level of education (*p* = 0.11) or smoking habits (*p* = 0.06). However, a significant association was found between asthma and nasal allergies (*p* < 0.001). The study concluded that asthma prevalence is significantly higher compared to most countries using the ECRHS questionnaire [30]. In 2016, Alqahtani reported a significant increase in allergic diseases over the last three decades, leading to higher morbidity and mortality in children and young adults. The study focused on determining the prevalence and risk factors of allergic diseases in 1700 Saudi school children from Najran in southwestern Saudi Arabia. The prevalence of asthma, allergic rhinitis and atopic dermatitis was 27.5%, 6.3% and 12.5%, respectively. Risk factors identified included male gender, consumption of fast food, nearby truck traffic and pet ownership. Sensitization to allergens was found in 42.5% of children, with grass pollen, cat fur and house dust mites being the most common. The study underlines the need for comprehensive intervention programs to reduce the burden of allergic diseases [31]. The similarities between asthma and allergic rhinitis (AR) have been known for many years. However, the immune system is the factor that plays the most important role in the pathophysiology of these two diseases. Further research revealed that the severity of asthma may trigger an inflammatory response in the upper airways, increasing the risk that AR will occur.

## 4. Cytokine Network in Asthma 

T helper cells have an essential function in modulating immunological responses. The network of cytokines, immune cells, and signaling pathways that make up the cytokine network in asthma is a dynamic and complicated system. Immune cells secrete tiny proteins called cytokines essential for controlling inflammation, immunologic responses, and intercellular communication. The various cytokines associated with asthma are listed in Table 2, along with information on how each contributes to the inflammatory and immunologic responses related to the disease. 

Cytokines are essential in mediating hyperreactivity, tissue remodeling, and airway inflammation associated with asthma. It is important to remember that our knowledge of the asthma cytokine network is constantly evolving, and new studies often reveal new subtleties and potential treatment targets. In addition, the profiles of different patients may change, leading to cytokine expression and response variations. As a result, tailored treatment plans are becoming increasingly crucial for asthma control [44]. 

### 4.1. Th_2_ Cytokines

In asthma, the immune system triggers inflammation that constricts the respiratory passages. This results in manifestations like difficulty breathing, coughing, wheezing, and a constricting feeling in the chest. Cytokines like IL-4, IL-5, and IL-13 often propel this immune reaction in asthma. The Th_2_ immune response is usually prevalent in asthma cases [45]. These Th_2_ cells emit a range of cytokines that amplify inflammation and the related symptoms. The following cytokines are involved in Th_2_ induction:

#### 4.1.1. Interleukin-4 (IL-4)

IL-4 plays a central role in the body’s allergic reactions, especially allergic asthma, and related diseases. This cytokine, produced primarily by Th_2_ cells, greatly influences how the immune system responds to allergens. One of the main functions of IL-4 is to induce naive T cells to develop into Th_2_ cells, which are tailored to trigger allergic reactions and secrete cytokines such as IL-5 and IL-13. These cytokines promote the arrival of eosinophils and stimulate mucus production in the airways. In addition, IL-4 directs B cells toward plasma cells that produce antibodies, particularly IgE. This specific antibody, IgE, is critical in allergic episodes, as it attaches to allergens and induces mast cells and basophils to release pro-inflammatory substances during subsequent allergen exposures [46]. Essentially, IL-4 causes B cells to produce these IgE antibodies, which in turn cause mast cells and basophils to recognize specific allergens. In addition, IL-4 is associated with the increased airway sensitivity that occurs in asthma, leading to increased bronchial constriction when exposed to allergens or other irritants. Along with IL-13, IL-4 increases mucus production in the airways, which can clog airways and make breathing difficult. Chronic exposure to IL-4 can even remodel the structure of the airways, increasing the persistent nature of asthma. 

#### 4.1.2. Interleukin-5 (IL-5)

IL-5 is of notable importance in the development of asthma, especially in cases of allergic asthma marked by eosinophilic inflammation. IL-5 is a key regulator of eosinophil growth, differentiation, activation, and survival. IL-5 is a potent stimulator of eosinophil production in the bone marrow and its migration into the airways and other tissues. In asthma, increased levels of IL-5 promote the accumulation of eosinophils in the airway walls and contribute to airway inflammation [47]. Eosinophils release various inflammatory mediators that can contribute to airway inflammation and tissue damage. These mediators can worsen airway hyperresponsiveness and bronchoconstriction, leading to asthma symptoms. Eosinophils, under the influence of IL-5, can also contribute to mucus production and secretion in the airways. Increased mucus can further obstruct the airways and impair lung function. Chronic exposure to eosinophils and their products can contribute to airway remodeling, which involves structural changes in the airway walls. This remodeling can lead to long-term changes in lung function and contribute to the chronicity of asthma [48]. IL-5 is often produced in conjunction with other Th_2_ cytokines such as IL-4 and IL-13. These cytokines work together to amplify the allergic response, leading to more severe inflammation and symptoms. 

#### 4.1.3. Interleukin-13 (IL-13)

IL-13 is a pivotal cytokine in the initiation and advancement of asthma, particularly in allergic asthma. It is intricately linked to the Th_2_ immune response and shares numerous functions with IL-4. Various immune cells, including Th_2_ cells, produce IL-13, which exerts significant impacts on both the airways and the immune system. IL-13 plays a crucial role as a primary instigator of airway inflammation in asthma, as noted in a study [49]. It promotes the recruitment of inflammatory cells, such as eosinophils, mast cells, and T cells, to the airways. This inflammation can lead to airway hyperresponsiveness and bronchoconstriction, resulting in asthma symptoms. IL-13 stimulates the production of mucus-secreting goblet cells in the airway epithelium. Increased mucus production can obstruct the airways, making breathing more difficult and contributing to asthma symptoms. IL-13 is associated with airway remodeling, a process characterized by structural changes in the airway walls, as observed in the study by Pope et al. 2001 [16]. This may lead to thickening of airway smooth muscle, increased deposition of extracellular matrix proteins, and changes in blood vessel architecture. These changes may play a role in persistent airway obstruction and deterioration of lung function over time. In addition, IL-13 has the potential to compromise the integrity of the airway epithelial barrier, which normally serves as a protective barrier against allergens and irritants. This barrier dysfunction can lead to increased allergen exposure and further inflammation. IL-13 promotes the production of IgE antibodies, like IL-4. IgE antibodies play a role in allergic responses by sensitizing mast cells and basophils to release inflammatory mediators upon allergen exposure. Chronic exposure to IL-13 can lead to the activation of fibroblasts and the production of collagen and other extracellular matrix components. This fibrotic response contributes to airway remodeling and structural changes.

#### 4.1.4. Interleukin-9 (IL-9) 

IL-9 is associated with the development of asthma, particularly its contribution to airway inflammation and increased sensitivity [50]. Originally recognized as a factor in promoting T-cell growth, the influence of IL-9 goes beyond simply supporting T-cell expansion. It plays a central role in attracting and activating various immune cells in the airways, such as eosinophils, mast cells, and T cells. Immune cells secrete inflammatory substances that play a role in airway inflammation and eventually lead to the manifestation of asthma symptoms. IL-9 stimulates the formation of mucus-producing goblet cells in the airway mucosa. An increase in mucus production can lead to airway obstruction and increase asthma symptoms. Immune cells secrete inflammatory substances that play a role in airway inflammation and eventually lead to the manifestation of asthma symptoms. T helper cells have an essential function in modulating immunological responses. IL-9 induces the proliferation and differentiation of goblet cells, responsible for mucus production, in the mucosa of the respiratory tract. Increased mucus secretion can lead to airway obstruction and exacerbate symptoms associated with asthma [51]. IL-9 increases airway sensitivity, a characteristic feature of asthma. This increased sensitivity results in more pronounced bronchoconstriction and restricted airflow when certain triggers occur. IL-9 plays an important role in the attraction and activation of eosinophils, which are often found in increased concentrations in the airways of asthmatics. Eosinophils are involved in allergic and inflammatory reactions and have the potential to damage tissues. IL-9 can stimulate mast cells, important immune cells that play a role in allergic reactions. When these mast cells are activated, they release histamine and other substances that cause airway constriction, mucus secretion and inflammation. IL-9 is a product of Th_9_ cells, a specific type of CD4+ T cell. This cytokine cooperates with other Th_2_ cytokines such as IL-4 and IL-13 and enhances the Th_2_ immune response commonly seen in asthma [16,35,49].

## 5. Cytokines of T Helper Cell Subsets

### 5.1. Interleukin-17 (IL-17) 

IL-17 is primarily associated with a type of immune response called the Th_17_ response. While Th_2_ responses dominate in allergic asthma, Th_17_ responses and IL-17 have also been implicated in certain asthma cases, particularly non-eosinophilic inflammation. IL-17 is often associated with neutrophilic inflammation, which is characterized by the recruitment and activation of neutrophils in the airways. This type of inflammation is distinct from eosinophilic inflammation, which typically occurs in allergic asthma. Neutrophilic inflammation can contribute to airway obstruction and airflow limitation. IL-17 is often associated with neutrophilic inflammation, which is characterized by the recruitment and activation of neutrophils in the airways [52]. This type of inflammation differs from eosinophilic inflammation, which typically occurs in allergic asthma. Neutrophilic inflammation can contribute to airway obstruction and airflow limitation [53]. Th_17_ asthma is a subtype of asthma associated with more severe, steroid-resistant cases and is characterized by neutrophilic inflammation driven by Th_17_ cells and the cytokine IL-17. Individuals at risk for Th_17_ asthma include those with obesity, exposure to pollutants, smoking, and respiratory infections. While Th_17_-driven asthma is generally non-allergic, there is a recognized Th_2_/Th_17_ overlap phenotype, where patients exhibit both Th_2_-mediated allergic responses (eosinophilic inflammation) and Th_17_-driven inflammation (neutrophilic). This overlap results in more severe asthma, often unresponsive to standard therapies, and highlights the need for treatments targeting both Th_2_ and Th_17_ pathways [54,55,56]. 

### 5.2. Tumor Necrosis Factor-Alpha (TNF-α) 

TNF-α is a proinflammatory cytokine that is crucial for systemic inflammation and belongs to the group of cytokines that trigger the acute phase response. While macrophages are the main producers, other cells such as CD4+ lymphocytes, Nk cells, neutrophils, mast cells, eosinophils, and neurons can also produce TNF-α [57]. TNF-α is associated with the onset of asthma, a long-term inflammatory lung disease characterized by bronchoconstriction, airway inflammation, and increased airway sensitivity [58]. The function of TNF-α in asthma is multifaceted and interferes with several processes. TNF-α can increase the display of adhesion molecules on endothelial cells, leading to increased entry of leukocytes into the airways. These leukocytes then release substances that increase inflammation. TNF-α can further stimulate the release of inflammatory substances, leading to bronchoconstriction and increased airway sensitivity. Because asthma manifests in different forms and phenotypes, the effect of TNF-α might differ among these variations. Moreover, TNF-α targeted treatment does not always prove to be effective for every asthma patient. Therefore, additional studies are essential to identify those who might respond positively to anti-TNF-α treatment. 

### 5.3. Interferon-Gamma (IFN-γ) 

IFN-γ is crucial for immune responses, mainly produced by activated T cells and natural killer cells and participates in various immune-related activities, such as macrophage activation, antigen presentation enhancement, and immune response regulation [59]. IFN-γ has both anti-inflammatory and pro-inflammatory effects, depending on the context. In asthma, a chronic lung disease marked by airway inflammation, bronchoconstriction, and airway hyperreactivity, the role of IFN-γ is multifaceted. Asthma can generally be divided into two, eosinophilic and non-eosinophilic [60]. IFN-γ is linked to the Th_1_ response, which is more common in non-eosinophilic asthma. In non-eosinophilic asthma, IFN-γ may serve as a protective function. The Th_1_ response can counteract the Th_2_ response, which is associated with eosinophilic inflammation. By encouraging a Th_1_ response, IFN-γ may reduce eosinophilic inflammation, which is connected to asthma exacerbations [44]. Additionally, IFN-γ has been demonstrated to suppress the release of pro-inflammatory mediators, such as histamine, from mast cells. However, excessive IFN-γ production may be harmful in asthma. Elevated levels of IFN-γ have been linked to airway remodeling, a characteristic of chronic asthma that causes structural changes in the airways. These alterations can result in reduced lung function and increased airway hyperresponsiveness. Researchers are still studying the role of IFN-γ in asthma, and there is ongoing interest in exploring how this cytokine interacts with other immune mediators in the context of asthma. Creating therapies that target IFN-γ or adjust the balance between Th_1_ and Th_2_ responses may be a potential direction for future asthma treatments. Nevertheless, these therapies must be meticulously developed to prevent unwanted side effects or inflammation exacerbation. 

## 6. Regulatory Cytokines 

Regulatory cytokines are crucial to the immune response in several health problems, such as asthma. Asthma is a persistent inflammatory ailment that impacts the pulmonary airways, resulting in manifestations such as wheezing, coughing, and dyspnea [43]. The immune system’s response to allergens or other triggers plays a central role in the development and exacerbation of asthma symptoms. Cytokines are small proteins that are released by immune cells and other cells in the body, and they help coordinate the immune response. There are several different types of cytokines, including pro-inflammatory cytokines and regulatory cytokines. Regulatory cytokines are responsible for modulating the immune response, which can help reduce inflammation and promote tissue repair in asthma [61]. The key regulatory cytokines that are involved in asthma include: 

### 6.1. Interleukin-10 (IL-10) 

IL-10 is a regulatory cytokine that can suppress inflammation and inhibit the activation and function of certain immune cells, such as T cells and macrophages. IL-10 can reduce the production of pro-inflammatory cytokines and other mediators of inflammation [62]. IL-10 can suppress the release of inflammatory cytokines such as TNF-alpha, IL-6, and IL-1 by immune cells like macrophages. This results in reduced inflammation. IL-10 can decrease the activation of immune cells like T cells, B cells, and macrophages. This reduces the immune response and, subsequently, inflammation. IL-10 can promote the differentiation and function of Tregs, which helps suppress inflammation and prevent autoimmune reactions [39]. IL-10 plays a critical role in counterbalancing the effects of proinflammatory cytokines, offering potential therapeutic benefits in asthma. 

### 6.2. Transforming Growth Factor-Beta (TGF-β) 

TGF-β is an important cytokine that regulates the immune system, suppresses the growth and activation of specific immune cells, and helps repair tissue damage. However, overproduction of TGF-β may be associated with airway changes related to asthma, resulting in structural shifts in the airways and increased resistance to airflow. It is also a significant mediator in immune response and tissue repair. TGF-β has been implicated in various aspects of the disease [36,63]. A characteristic feature of chronic asthma is changes in the structure of the airway walls; a phenomenon called airway remodeling. This transformation includes thickening of the subepithelial region, enlargement of smooth muscle, and formation of new blood vessels. TGF-β plays a role in this process by promoting fibroblast growth and the accumulation of extracellular matrix components, which leads to thickening of the subepithelial layer. In addition, TGF-β can also influence the proliferation of smooth muscle cells. In asthma, Epithelial–Mesenchymal Transition (EMT) is thought to be a factor in airway changes or remodeling that can lead to thickening of airway walls, which impairs airflow and reduces lung efficiency [64]. TGF-β plays a crucial role in this process by inducing these epithelial cells to undergo EMT, causing them to lose their original properties and take on mesenchymal characteristics. This not only leads to an increase in the production of extracellular matrix components, but also promotes the cells’ ability to migrate and invade. TGF-β plays an important role in airway remodeling, a defining feature of chronic asthma. Its promotion of EMT can lead to the thickening of airway walls (known as subepithelial fibrosis). In addition, TGF-β can also promote fibroblast growth and transformation, which contributes to airway fibrosis. The interrelationship between EMT and TGF-β in asthma underscores how cell transformation and regulatory factors intertwine, exacerbating the complexity of the disease [41,65]. A deeper exploration of this link may pave the way for better treatment strategies in the future.

### 6.3. Interleukin-35 (IL-35)

IL-35, a newly identified regulatory cytokine, is characterized by its ability to inhibit T-cell activation and reduce the release of inflammatory cytokines, making it a potential asset in controlling immune responses in asthma [66]. Its immunosuppressive functions have led to its exploration as a therapeutic target for inflammatory and autoimmune diseases, including asthma. However, the complex role of regulatory cytokines in asthma requires a deeper understanding of their specific mechanisms to develop targeted and effective treatments for this disease.

## 7. Dysregulated Immune System in Asthma 

Dysregulated immune system is a condition where the immune system erroneously targets and harms the body’s tissues, cells, or organs. Although asthma is primarily recognized as a chronic respiratory ailment characterized by airway inflammation and constriction, there is ongoing research and conjecture regarding potential autoimmune mechanisms that might influence specific aspects of asthma [67]. In many autoimmune diseases, specific autoantibodies often serve as unique markers for the disease. Recently, there has been growing interest in a possible link between autoantibodies and asthma, a primary inflammatory disease of the airways [68,69,70,71]. Some research suggests that people with asthma may have autoantibodies, which in some cases may play a role in the underlying mechanisms of the disease. It has been observed that certain asthma patients have autoantibodies that target bronchial epithelial cells. Such autoantibodies could contribute to the epithelial damage and dysfunction typical of asthma. In addition, there are data to suggest that these autoantibodies may be more common or have a greater impact in patients with severe asthma than in patients with milder forms of the disease. 

Th_17_ cells are an immune cell subtype that participates in autoimmune reactions. They generate cytokines that have an inflammatory effect. Th_17_ cells and the cytokines they produce are in elevated concentrations in the airways of asthmatic patients, especially those with severe or steroid-resistant conditions [72,73,74]. Treg cells are critical in regulating the immune response and preventing autoimmune reactions. Dysfunction of these cells may play a role in the development of asthma and its subsequent severity. Dysfunction of the immune system plays a vital role in the development and progression of asthma. 

The phenomenon of immunological dysregulation in asthma refers to an imbalance in the immune response, resulting in an enhanced inflammatory response and increased airway sensitivity [12]. An enhanced Th_2_ immune response is associated with a significant immunological imbalance associated with asthma. Th_2_ cells, which are a subset of T cells, are responsible for the release of several cytokines such as IL-4, IL-5, and IL-13. These cytokines promote the development of allergic inflammation, the production of mucus and the accumulation of eosinophils in the airways, leading to the characteristic symptoms of asthma. Asthma is characterized by dysregulation of the immune system, which leads to an increased presence of eosinophils in the airways, causing tissue damage and inflammation. Allergic asthma is often associated with this form of inflammation [27]. 

Over time, an imbalance in the immune system can cause structural changes in the airways, a phenomenon called airway remodeling. This phenomenon includes thickening of airway walls, increased mucus production, and changes in airway smooth muscle cells. Such changes can lead to persistent airway obstruction and decreased lung capacity. Cytokines play a crucial role in immune signaling. In asthma, there is an imbalance in cytokine production. For example, increased IL-4, IL-5, and IL-13 promote the Th_2_-mediated allergic response, while reduced levels of regulatory cytokines like IL-10 lead to inadequate control of inflammation [75,76,77]. The innate immune system, which provides rapid but nonspecific responses to infections and antigens, is also implicated in asthma. Pattern recognition receptors (PRRs) on immune cells recognize specific molecular patterns in pathogens and can trigger inflammation [78,79,80,81]. In asthma, PRRs may contribute to excessive inflammation even in the absence of infection. On the other hand, IgE is an antibody class involved in allergic responses [82,83]. In asthma, immune dysregulation can lead to the production of IgE antibodies against harmless substances, triggering allergic reactions and contributing to airway inflammation. Mast cells, basophils, and other immune cells play a role in asthma by releasing inflammatory mediators like histamine and leukotrienes. This immune cell activation is dysregulated in asthma, leading to increased bronchoconstriction and inflammation [84]. 

## 8. Challenges in Therapeutics, Current Research, and Future Directions 

Asthma, a long-standing respiratory disease, poses several treatment problems (Table 3). These range from the multifaceted nature of the disease to complications such as steroid resistance and the effects of prolonged medication use. Many of the available treatments focus largely on relieving symptoms rather than addressing the causes, leaving some patients to struggle with severe flare-ups. But the outlook is promising. 

Current research focuses on biologics, with an emphasis on monoclonal antibodies that target specific inflammatory agents. Cutting-edge techniques such as bronchial thermoplasty are also gaining attraction [103,104]. The advent of personalized medicine, aided by genomics and proteomics, points to tailored interventions for individual patients. Future treatments have the potential for advanced drug delivery systems, regenerative therapies, and a harmonious blend of traditional medicine and lifestyle changes [104,105]. By addressing environmental catalysts and pooling resources in collaborative research, asthma treatment could undergo a revolutionary change. In-depth studies are currently underway targeting biologic treatments that focus on specific asthma-related pathways. For example, monoclonal antibodies targeting interleukins and other inflammation-inducing molecules are under scrutiny. Breakthroughs in genomics and biomarker investigation are setting the stage for individualized asthma therapies. Identifying biomarkers that match treatment outcomes enables fine-tuning of therapeutic interventions for each patient. Tools such as CRISPR/Cas9 offer exciting possibilities. They could potentially alter genes associated with asthma, introducing a breakthrough strategy to address the causes of the disease [106]. The intricate relationship between the lung microbial ecosystem and the onset and progression of asthma is also being studied, which could lead to innovative treatments that target airway microbiota. Empowering patients through education and self-regulation may enable them to better manage their asthma and minimize the risk of an outbreak. Digital healthcare, with its many smartphone applications and wearable technologies, can support self-regulation, ensure consistent medication adherence, and provide early warnings of an asthma attack occurrence [107]. The development of effective combination therapies that address multiple facets of the complex nature of asthma could lead to better management with fewer side effects. In addition, early interventions could alter the course of asthma, potentially curbing or mitigating its effects in later years. 

## 9. Conclusions

The complicated nature of asthma, characterized by a range of symptoms and triggers, has been the subject of extensive research. Recent revelations about allergic biomarkers and dysregulated immune system have greatly expanded our understanding of the disease. Allergic biomarkers, such as specific IgE antibodies and eosinophil counts, shed light on the allergic aspects of asthma. At the same time, the detection of autoinflammatory, in the immune system attacks its own during auto-inflammation response. This suggests that some asthmatics may struggle with both external allergens and internal autoimmune responses. In such individuals, the disease may take a more complex course that may not respond effectively to treatments that focus exclusively on the allergic dimension. From a therapeutic perspective, this dual perspective opens possibilities for treatments that combine conventional symptom management with methods that target the autoimmune side, such as monoclonal antibodies. Early identification of these markers could also lead to preventive measures by identifying high-risk individuals before they become symptomatic and allowing timely interventions. The intertwining of allergic biomarkers and dysregulated immune system in asthma symbolizes a new diagnostic and therapeutic frontier. This promises more precise and personalized treatment approaches and could reduce the global impact of this common respiratory disease.

## Figures and Tables

**Table 1:** Allergic markers in Asthma.

Allergic Marker	Role in Asthma	References
IgE (Immunoglobulin E)	IgE is critical in triggering allergic reactions and often has elevated levels in individuals with allergic asthma. Allergic asthma is usually triggered by environmental antigens such as dust, pollen, mold, and animal dander.	[18]
Eosinophils	Elevated levels of eosinophils are a sign of allergic inflammation often associated with asthma conditions. Eosinophilic asthma is recognized as a unique subtype within the broader category of asthma illnesses. This subtype is characterized by a distinct profile of inflammatory cells, specifically eosinophils, that invade the respiratory passages.	[19]
FeNO (Fractional exhaled nitric oxide)	Fractional exhaled nitric oxide (FeNO) serves as a non-invasive indicator of inflammation in asthma patients and provides a means of tracking airway inflammation. Despite its utility, the individual variability in FeNO levels makes it difficult to tailor treatment plans based solely on this marker. Elevated FeNO levels can indicate allergic inflammation in the airways.	[20]
Histamine	Histamine, which is released by mast cells during allergic reactions, plays a key role in airway obstruction by triggering smooth muscle contraction, increasing bronchial secretion, and causing swelling of the airway mucosa. Due to its short half-life, ranging from minutes to hours, it acts rapidly and leads to immediate effects such as bronchoconstriction, vasodilation, and mucus secretion. However, its influence on long-term airway inflammation is limited by this short duration of action. During an allergic episode, the release of histamine is a major factor in bronchoconstriction, which narrows the bronchi and obstructs airflow.	[21,22]
Cytokines	Th_2_ cytokines, including IL -4, IL -5, and IL -13, often have elevated baseline levels in individuals with allergic asthma, underscoring their role in the immune response. These elevated cytokine levels are associated with a greater number of eosinophils in both the airways and the bloodstream, as well as increased serum levels of allergen-specific IgE, commonly noted in allergic asthma cases.	[13]
Mast Cells	In individuals with asthma, mast cells are strategically positioned in different parts of the lung and play a crucial role as effectors and controllers in different asthma subtypes. Mast cells release mediators that stimulate a broad range of cellular activities in both the innate and adaptive immune systems, particularly in relation to inflammation within the airways. During allergic reactions, mast cells take on a critical role by producing histamine and other mediators that contribute to the allergic response.	[23,24]
Leukotrienes	Leukotrienes are lipid-based compounds that are essential in both acute and chronic inflammation, as well as in allergic reactions. These lipid molecules specifically contribute to airway inflammation and are notably significant in the context of allergic asthma. Leukotrienes, have a longer half-life of several hours, which contributes to their more sustained role in promoting bronchoconstriction, vascular permeability and inflammation in asthma.	[25]
T-helper cells (Th_2_)	T helper _2_ (Th_2_) cells were previously considered central to regulating type 2 immune responses in asthma, especially due to their role in allergic reactions and in boosting the production of IgE antibodies. Nevertheless, the importance of Th_2_ cells in this scenario has diminished with the discovery of other robust sources of type 2 cytokines and additional innate mediators of inflammation.	[13]
Allergen-specific IgG4	In individuals sensitized to allergens, exposure primarily increases the production of immunoglobulin E (IgE) antibodies and activates T cells. On the other hand, allergen-specific immunotherapy (AIT) is effective in promoting the production of allergen-specific IgG4 antibodies and decreasing T-cell activity. In both cases, the complexes that result from the interaction between allergens and antibodies are crucial for controlling the subsequent allergen-specific immune responses.	[26]

**Table 2:** The cytokines in asthma.

Cytokine	Role in Asthma	References
IL-4	IL-4 is instrumental in the differentiation of T-helper 2 (Th_2_) cells and plays a critical role in the production of IgE antibodies.	[32]
IL-5	IL-5 is significantly involved in the formation and persistence of eosinophils. It is also key in promoting inflammation that involves eosinophils, commonly referred to as eosinophilic inflammation.	[33]
IL-13	IL-13 shares functional similarities with IL-4 and plays a significant role in the creation of mucus within the airways. It also contributes to heightened sensitivity and responsiveness of the airways, often observed in asthma.	[34]
IL-9	IL-9 is important for enhancing the activity of mast cells, which are key players in allergic reactions. It also plays a role in mucus production in the respiratory tract.	[35]
IL-17	IL-17 plays a central role in inflammation associated with neutrophil involvement. In severe asthma, cytokines and immune elements derived from T helper 17 (TH_17_) cells are inextricably linked to the migration of neutrophils into the airways.	[36]
IL-33	IL-33 stimulates innate immune cells such as basophils, eosinophils, and mast cells, thereby promoting type 2 inflammation. It plays a role in initiating Th2 immune responses and is linked to airway inflammation.	[37]
IL-6	IL-6 serves as a broad-spectrum pro-inflammatory cytokine and may play a role in the acute phase response observed in cases of severe asthma.	[38]
IL-10	IL-10 serves as a regulatory cytokine with anti-inflammatory properties that help modulate the immune system’s response. It plays a pivotal role in managing allergic reactions and asthma symptoms by maintaining immunological balance.	[39]
IL-25	IL-25 is involved in activating and differentiating dendritic cell populations, thereby facilitating a previously undiscovered innate immune pathway. This pathway is thought to enhance Th_2_ cytokine responses, which are often implicated in airway inflammation seen in asthma.	[40]
TGF-β	TGF-β plays a role in the restructuring and fibrosis of airways and has a multifaceted function in the regulation of the immune system.	[41]
TNF-α	TNF-α acts as a proinflammatory cytokine and contributes to airway inflammation, a typical feature of asthma. In asthma, TNF-α could serve to attract neutrophils and eosinophils due to its pro-inflammatory properties.	[42]
IFN-γ	In asthma, Th_1_ cells and their associated cytokine, IFN-γ, play a complex role that involves a range of immune responses. They have the potential to counterbalance Th2-driven activities,	[43]

**Table 3:** Summary of current asthma drug therapies.

Type of Drug Therapy	Description	References
Acting Beta-2 Agonists (SABA)	Bronchodilators such as albuterol (salbutamol) and levalbuterol (levosalbutamol) are fast-acting drugs that are primarily used to provide rapid relief during acute asthma attacks. These medications work by relaxing the smooth muscle surrounding the airways by stimulating beta-2 adrenergic receptors, resulting in bronchial dilation and improved airflow. Albuterol and levalbuterol are often referred to as rescue inhalers as they work quickly, usually within minutes, to provide immediate relief from symptoms such as wheezing, shortness of breath and chest tightness. However, their effect is short-lived, so they are not suitable for long-term asthma control. Frequent or excessive use of these medications can lead to habituation, which reduces their effectiveness over time. Therefore, these short-acting bronchodilators are recommended for occasional use in emergencies rather than for the daily treatment of asthma.	[85,86]
Long-Acting Beta-2 Agonists (LABA)	Long-acting beta-2 agonists (LABAs) are bronchodilators for treating chronic respiratory diseases such as asthma and chronic obstructive pulmonary disease (COPD). By stimulating the adrenergic beta-2 receptors in the lungs, they relax the smooth muscles, resulting in prolonged bronchodilation of up to 12 h or more. Examples are salmeterol and formoterol, often combined with inhaled corticosteroids to better control symptoms. LABAs offer advantages such as reduced dosing frequency, better long-term disease management, and improved quality of life for patients. However, they are not suitable for acute symptom relief, can cause side effects such as palpitations and tremors, and prolonged use can lead to tolerance or increased cardiovascular risks. In addition, the use of LABAs without corticosteroids in asthma patients has been associated with an increased risk of mortality, underlining the importance of appropriate combination therapy.	[87,88]
Inhaled Corticosteroids	Inhaled corticosteroids (ICS) are a cornerstone of the long-term treatment of chronic respiratory diseases such as asthma and chronic obstructive pulmonary disease (COPD). They act on airway inflammation, the main cause of respiratory symptoms, by inhibiting inflammatory cells and blocking the release of cytokines to reduce swelling and mucus production. By controlling inflammation, ICS helps to prevent asthma attacks, improve lung function, and reduce the need for emergency medication. Common examples include budesonide, fluticasone, and beclomethasone, which are administered via inhalers and act locally in the lungs, minimizing the systemic side effects of oral corticosteroids. While highly effective for long-term control, they are not suitable for immediate symptom relief and are often combined with bronchodilators such as LABAs for comprehensive disease management. ICS offers numerous benefits, including fewer exacerbations, better quality of life, and fewer emergency admissions, but can cause local side effects such as oral thrush or hoarseness if the inhalation technique is not used correctly. High doses or prolonged use can lead to systemic effects such as suppression of adrenal function or growth in children, although these risks are lower compared to systemic corticosteroids and require careful monitoring during high-dose or long-term therapy.	[89,90,91,92]
Oral Corticosteroids	Oral corticosteroids such as prednisone and methylprednisolone are powerful systemic anti-inflammatory medications that are mainly used to treat severe exacerbations of diseases such as asthma, COPD, and autoimmune diseases. These drugs suppress the immune response and reduce inflammation throughout the body, providing rapid and comprehensive relief during acute flare-ups. They are often used in short episodes to quickly control symptoms and avoid complications such as hospitalization but can also be prescribed long-term in smaller doses for chronic conditions. One of the advantages of oral corticosteroids is that they are fast-acting and can relieve inflammation in multiple tissues and organs, making them indispensable in emergencies or when local treatments are not sufficient. However, their systemic effect leads to significant disadvantages, especially with prolonged use, as they can cause serious side effects such as weight gain, diabetes, hypertension, osteoporosis, and adrenal suppression. These risks arise from their effects on various metabolic and hormonal pathways, leading to complications such as increased blood sugar, increased blood pressure, bone density loss, and impaired natural corticosteroid production. While oral corticosteroids are essential for the control of severe symptoms, their long-term use must be carefully managed to minimize adverse effects.	[93,94]
Leukotriene Modifiers	Leukotriene modifiers such as montelukast and zafirlukast are medications that reduce airway inflammation and bronchoconstriction by blocking leukotrienes, which are involved in allergic and inflammatory reactions. These drugs are often used to treat conditions such as asthma and allergic rhinitis. An advantage of leukotriene modifiers is that they provide an oral, non-steroidal option for controlling asthma symptoms, making them suitable for long-term use, particularly in patients with exercise-induced bronchoconstriction. However, a notable drawback is the potential for neuropsychiatric side effects such as mood swings, depression, and insomnia, which may affect a subset of patients. For example, montelukast, although effective, has been associated with such side effects in some cases. Despite these risks, leukotriene modifiers remain a valuable tool in the treatment of respiratory diseases, especially when corticosteroids or beta-agonists are not sufficient.	[95,96]
Theophylline	Theophylline, a methylxanthine derivative, is a bronchodilator used to treat asthma and COPD by relaxing bronchial muscles by inhibiting phosphodiesterase and increasing cyclic AMP levels, improving respiratory function. Theophylline also has a mild anti-inflammatory effect, making it a helpful adjunct therapy for poorly controlled asthma or COPD patients. Theophylline is taken orally, which improves patient compliance and provides prolonged bronchodilation when administered in controlled-release forms. However, it has a narrow therapeutic index, which increases the risk of side effects such as nausea, vomiting, cardiac arrhythmias, and seizures, especially at high blood levels, necessitating regular monitoring. Interactions with other medications and factors such as age and smoking also affect metabolism and make it difficult to use. Despite these problems, it remains an option for patients who do not respond to first-line therapies such as inhaled corticosteroids or long-acting beta-agonists. However, careful monitoring is required to weigh up the benefits and risks.	[97,98]
Mast cell stabilizers	Mast cell stabilizers, particularly Cromolyn Sodium, are crucial in managing allergic conditions and asthma by preventing mast cell degranulation and the subsequent release of inflammatory mediators. As prophylactic agents, they are primarily used to avert symptoms rather than treat acute episodes. Cromolyn Sodium is especially beneficial for individuals at risk of exercise-induced asthma, allowing them to engage in physical activities without triggering an inflammatory response. However, its effectiveness is somewhat limited compared to inhaled corticosteroids (ICS), the gold standard for controlling inflammation in asthma management. One of the notable challenges with Cromolyn Sodium is the requirement for frequent dosing, as it does not provide sustained relief, necessitating careful adherence to the dosing schedule to maintain its preventive benefits. Furthermore, while it is generally well-tolerated and safe for long-term use, its efficacy in treating acute asthma attacks is limited, making it less suitable for immediate relief. Overall, while Cromolyn Sodium has advantages, such as its safety profile and its role in prevention, its efficacy and dosing frequency limitations highlight the importance of considering other treatment options for comprehensive asthma management.	[99,100]
Monoclonal Antibodies	Monoclonal antibodies such as omalizumab represent a class of biologics targeting immune components such as immunoglobulin E (IgE) or interleukin-5 (IL-5) to relieve inflammation and allergic reactions. Omalizumab, for example, binds to free IgE and prevents it from interacting with its receptor on mast cells and basophils, thereby reducing allergic reactions and inflammation, making it particularly effective in treating severe allergic asthma. Other biologics that target IL-5, such as mepolizumab, reduce the activity of eosinophils, which are the main drivers of inflammation in conditions such as eosinophilic asthma. These biologics have been shown to be effective in reducing the frequency of exacerbations and improving overall control in patients with severe allergic diseases that do not respond to conventional therapies. However, despite their therapeutic benefits, these treatments come at a high cost and are administered by injection, which carries a severe but rare risk of anaphylaxis. Due to their targeted mechanisms of action, biologics such as omalizumab have become an important option in treating refractory allergic diseases, although they require careful monitoring of patients.	[101,102]

## Data Availability

The data supporting the conclusions of this review article can be found within the article itself.
